# Giant 90-mm Ossified Stylohyoid Chain Associated with Restricted Mouth Opening

**DOI:** 10.3390/diagnostics16193197

**Published:** 2026-10-01

**Authors:** Jakub Bargiel, Michał Gontarz, Tomasz Marecik, Krzysztof Gąsiorowski, Grażyna Wyszyńska-Pawelec

**Affiliations:** Department of Cranio-Maxillofacial Surgery, Jagiellonian University Medical College, 30-688 Cracow, Poland; michal.gontarz@uj.edu.pl (M.G.); tomasz.marecik@uj.edu.pl (T.M.); krzysztof.gasiorowski@uj.edu.pl (K.G.); grazyna.wyszynska-pawelec@uj.edu.pl (G.W.-P.)

**Keywords:** Eagle syndrome, ossified stylohyoid chain, styloid process, limited mouth opening, styloidectomy, computed tomography angiography, internal jugular vein narrowing, orofacial pain

## Abstract

A 62-year-old man was referred with a six-month history of right submandibular pain followed by dysphagia. At presentation, maximal interincisal opening was 25 mm. Computed tomography angiography demonstrated an ossified right stylohyoid chain extending from the skull base to the hyoid region. It measured 90 mm in length and 17.5 × 10.5 mm at maximal cross-section. The chain contacted the external carotid artery, lay 1.1 mm from the internal carotid artery, and was associated with focal narrowing of the internal jugular vein between the chain and the transverse process of the atlas, with preserved venous patency. Because of restricted mouth opening, tracheal intubation required combined fiberoptic and videolaryngoscopic guidance. Under general anesthesia and neuromuscular blockade, maximal passive interincisal opening, measured with a ruler by the operating surgeon, remained 25 mm before resection and increased immediately to 45 mm after transcervical resection using a diamond burr. Distally, the chain was attached to the lesser horn of the hyoid bone by a residual stylohyoid ligament, which was released without hyoid bone osteotomy. The two excised fragments measured 80 mm in combined length, with an approximately 10-mm proximal segment intentionally retained. Pain was resolved, and maximal interincisal opening was 42 mm at six months. This case highlights the value of vascular imaging, precise definition of stylohyoid measurements, and consideration of Eagle syndrome in unexplained restriction of mouth opening.

**Figure 1:**
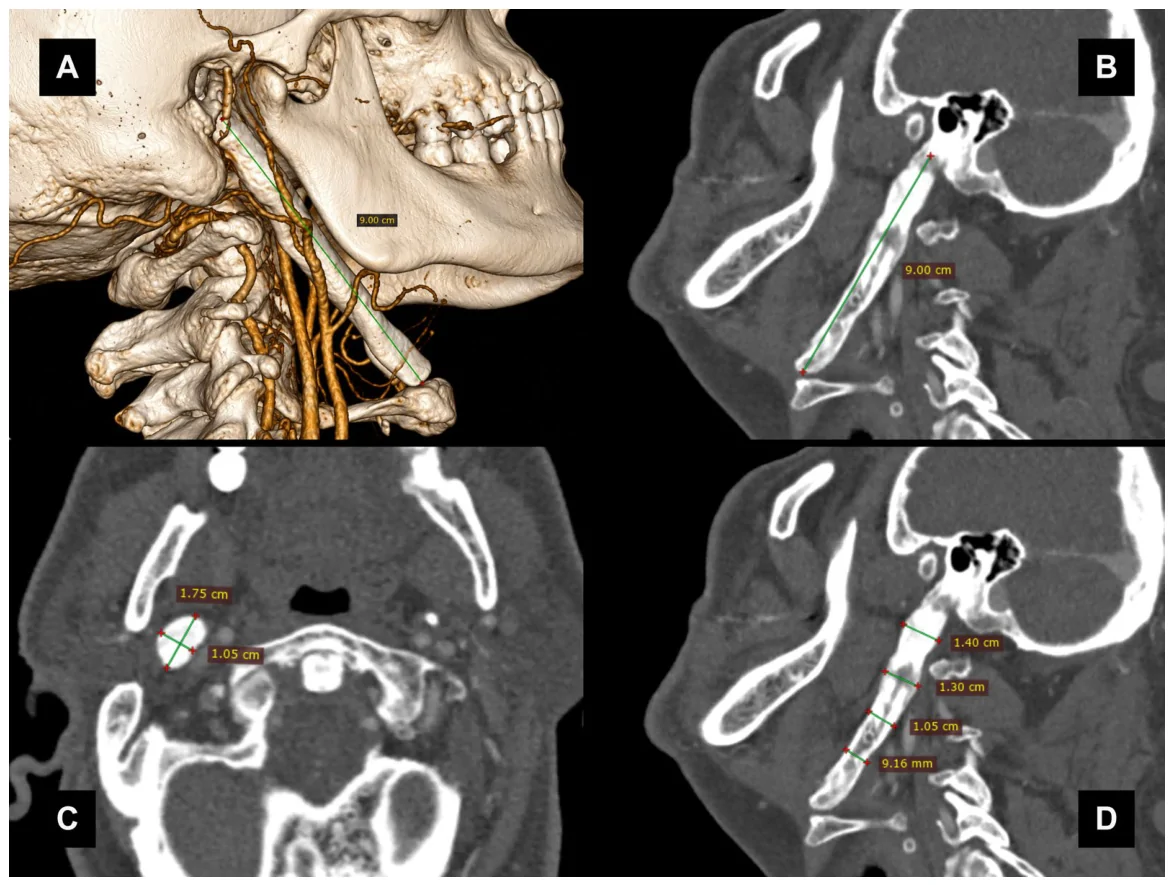
Preoperative computed tomography angiography (GE Healthcare Discovery CT750 HD) of a 62-year-old man with an ossified right stylohyoid chain. (**A**) Three-dimensional reconstruction demonstrating the ossified stylohyoid complex and its relationship to the major cervical vessels. The radiological length of the complex was 90 mm. (**B**) Multiplanar reconstruction along the longitudinal axis confirming a length of 90 mm. (**C**) Axial image at the level of maximal transverse dimensions, demonstrating a cross-section of 17.5 × 10.5 mm. (**D**) Longitudinal reconstruction demonstrating the width of the ossified complex at four levels, ranging from 14.0 mm near the skull base to 9.2 mm distally. The styloid process projects from the inferior surface of the temporal bone and is connected to the hyoid bone by the stylohyoid ligament. Although a length greater than approximately 30 mm is generally considered elongated, styloid process length alone does not determine the presence or severity of symptoms [1,2]. Six months before referral, the patient developed persistent right submandibular pain and was evaluated by an otolaryngologist. The symptoms were initially treated as tonsillitis, but antibiotic therapy was ineffective and subsequent otolaryngological examination did not confirm active tonsillar inflammation. During the following months, the pain persisted, and approximately five weeks before referral, the patient also developed dysphagia. On admission to our department, maximal interincisal opening was 25 mm. The duration of the restricted mouth opening could not be established, as the patient had not previously perceived it as abnormal. Given the marked restriction of mouth opening, alternative local causes were considered before relating this finding to the stylohyoid complex. Potential causes included temporomandibular joint disease, coronoid process abnormalities, odontogenic or inflammatory pathology, and sequelae of previous trauma or surgery. Clinical examination and CT did not identify osseous temporomandibular joint abnormalities, coronoid process abnormalities, or odontogenic or inflammatory pathology sufficient to explain the degree of restriction. There was no history of trauma or surgery in the affected region. CT assessment does not exclude temporomandibular joint disc displacement or other soft-tissue internal derangement. However, the immediate increase in passive opening after resection, without temporomandibular joint manipulation, argues against disc-related locking as the primary cause. The limitation of mouth opening was therefore considered in the context of the patient’s remaining clinical findings rather than regarded in isolation as a manifestation of Eagle syndrome. There was no facial or cervical swelling. His medical history was notable for myocardial infarction, arterial hypertension, and acute promyelocytic leukemia in 2014. There was no history of renal disease. The diagnostic work-up focused on a presumed local enthesopathic process; targeted laboratory assessment of mineral metabolism, including serum calcium, phosphate, and parathyroid hormone, was not performed. Therefore, a systemic metabolic contribution cannot be excluded. The reported medical history does not establish a cause of the stylohyoid ossification. Clinical examination revealed a hard, elongated structure corresponding to the ossified stylohyoid complex, palpable both intraorally and through the neck. Imaging demonstrated an ossified stylohyoid chain extending from the skull base to the hyoid region, without pseudoarticulation, corresponding to a Langlais type I morphology [3]. Intraoperatively, its distal attachment to the lesser horn of the hyoid bone was found to comprise a residual stylohyoid ligament rather than osseous fusion with the hyoid bone. The left stylohyoid complex was also elongated, measuring 46 mm, but was asymptomatic, consistent with our series, in which three-quarters of patients had an elongated complex on the asymptomatic side [4]. Notably, the right-sided ossified complex showed no marked medial angulation. Length, angulation, and cross-sectional dimensions describe different aspects of stylohyoid morphology [5]. In this patient, the 90-mm length and the maximal cross-section of 17.5 × 10.5 mm are anatomical observations; their independent contributions to symptoms cannot be determined from a single case. These dimensions should not be interpreted as validated thresholds for symptomatic disease. Cross-sectional dimensions are rarely reported in descriptions of exceptionally long stylohyoid complexes. Reporting both length and cross-sectional dimensions may improve comparison between such cases. The long, ossified chain, tethered distally to the hyoid bone by the residual ligament, could plausibly restrict hyoid excursion and alter tension in the suprahyoid and adjacent soft tissues during mandibular depression. This is a proposed mechanism, not a demonstrated point of obstruction. Static CTA and three-dimensional reconstruction do not, by themselves, demonstrate direct impingement against the mandibular ramus during opening. The intraoperative change is therefore interpreted as supporting a mechanical contribution rather than proving a specific anatomical mechanism. The differential diagnosis of cervicofacial pain and dysphagia also includes temporomandibular disorders, myofascial pain, glossopharyngeal or trigeminal neuralgia, tonsillar or deep-neck infection, salivary disease, and pharyngeal or skull-base neoplasia [6]. In clinically selected patients with otherwise unexplained neck pain and dysphagia, panoramic radiography can provide an initial assessment of stylohyoid elongation or ossification [7]. It does not establish that the radiographic finding causes the symptoms and does not assess vascular relationships. CT with multiplanar and three-dimensional reconstruction better defines the anatomy; CTA is particularly useful when vascular involvement is suspected or vascular relationships need to be mapped before surgery [2,8]. Persistent dysphagia requires appropriate clinical evaluation even when panoramic imaging shows an elongated stylohyoid complex.

**Figure 2:**
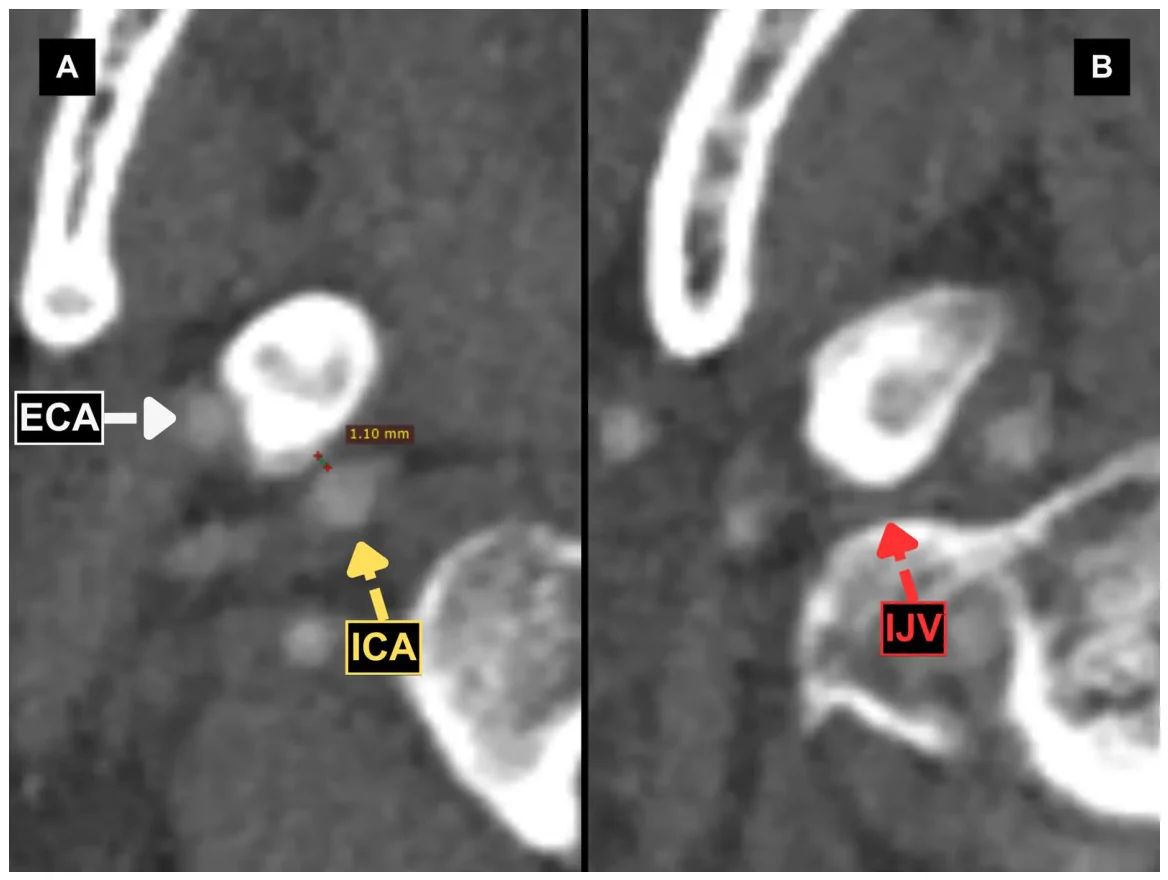
Relation of the ossified stylohyoid chain to the vessels of the neck on computed tomography angiography (CTA). (**A**) Axial section at the level of the second cervical vertebra, where the complex lies closest to the vessels. It is in direct contact with the right external carotid artery (ECA; white arrow) and lies 1.1 mm from the right internal carotid artery (ICA; yellow arrow). The green line with red crosses indicates the 1.1 mm measurement. (**B**) Axial section at the level of C1 demonstrates focal narrowing of the right internal jugular vein (IJV; red arrow) as it passes between the ossified complex and the transverse process of the atlas (red arrow). The vein remained patent, with preserved contrast opacification and normal caliber cranial and caudal to the narrowed segment. The patient had no facial or neck swelling. No additional symptoms were reported beyond the pain, dysphagia, and restricted mouth opening described above. No targeted assessment for intracranial hypertension, funduscopic examination, or dedicated functional venous outflow investigation was undertaken. Extrinsic narrowing of the upper internal jugular vein is common in unselected patients and is often related to the styloid process or adjacent structures. Focal narrowing on a static CTA image alone does not establish clinically significant venous outflow obstruction [9]. Conversely, preserved opacification and the absence of facial or neck swelling do not establish normal cerebral venous drainage. These observations alone cannot determine the hemodynamic significance of the narrowing. Vascular complications of Eagle syndrome include carotid artery dissection and cerebral ischemic events [6,10]. Styloid-related IJV compression can also cause symptomatic venous outflow obstruction and secondary intracranial hypertension [11]. Reported manifestations include headache, pulsatile tinnitus, visual disturbances, and papilledema [12]. These complications justify attention to vascular relationships, but their occurrence in other patients does not establish clinically significant obstruction in the present case. Clinical findings should guide any dedicated venous and neuro-ophthalmological assessment [11,12]. CTA was particularly valuable in this case because it demonstrated the close relationship of the ossified stylohyoid complex to the carotid arteries and internal jugular vein and allowed preoperative planning of the extent of safe surgical exposure and resection [8].

**Figure 3:**
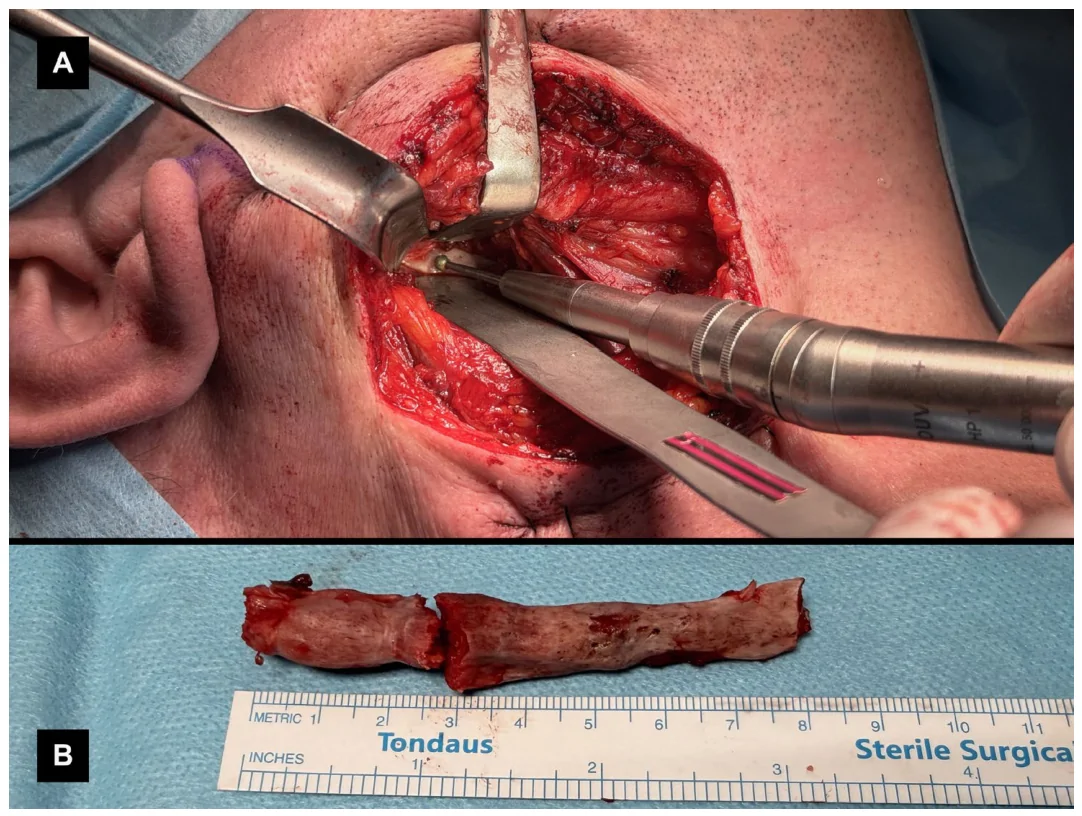
Surgical removal of the ossified stylohyoid chain. (**A**) Intraoperative view during osteotomy with a diamond burr near the skull base. The proximal osteotomy was positioned approximately 10 mm below the skull base, leaving a short proximal segment in situ to avoid dissection immediately adjacent to the stylomastoid foramen and the neurovascular structures at the skull base. (**B**) The combined length of the two excised fragments was 80 mm. Radiological length and combined specimen length were measured separately: the former included the proximal segment intentionally retained near the skull base. The procedure was performed under general anesthesia. Preoperative maximal interincisal opening was limited to 25 mm, and airway management required combined fiberoptic and videolaryngoscopic guidance. A transcervical approach was selected, with the incision placed within a natural skin crease below the angle of the mandible. After division of the platysma, blunt dissection continued toward the skull base. We routinely use a minimally invasive cervical approach through a short incision for elongated styloid processes [8]. In the present case, however, the dimensions and marked thickness of the ossified complex required broader exposure. Division with rongeurs or similar instruments was avoided because fragmentation of such a thick ossified structure could have been difficult to control in close proximity to the carotid vessels and the internal jugular vein. A wider transcervical exposure was therefore used, and osteotomy was performed with a diamond burr under continuous irrigation. After identification and protection of the carotid vessels, the ossified complex was additionally divided at its widest portion. Following osteotomy, the distal fragment was detached from the lesser horn of the hyoid bone by releasing the residual stylohyoid ligament. This distal separation was straightforward and did not require osteotomy. The distal ossified fragment was removed without inclusion of hyoid bone in the specimen; only the approximately 10-mm proximal segment was intentionally retained. The two fragments were removed under direct vision. Relevant neurovascular structures in this region include the facial nerve near the skull base, the internal and external carotid arteries, the internal jugular vein, and the lower cranial nerves within the retrostyloid compartment. A transoral approach was considered less appropriate because the restricted mouth opening and the close relationship of the ossified complex to the adjacent vascular structures would have limited both exposure and direct control of the operative field [13]. The procedure lasted two hours, blood loss was negligible and no vessel required ligation. After induction of general anesthesia and administration of neuromuscular blocking agents, but before resection of the ossified stylohyoid chain, maximal passive interincisal opening remained 25 mm, consistent with the preoperative awake measurement. Both intraoperative measurements were performed by the operating surgeon using the same ruler-based method between the incisal edges of the upper and lower incisors, with the patient under neuromuscular blockade. Maximal passive opening was assessed gently, without forceful mandibular manipulation or stretching during either assessment. No mouth gag, mandibular stretching, or other manipulation of the mandible was applied between the two measurements, and the transcervical procedure did not involve the oral cavity. Immediately after removal of the ossified stylohyoid chain, maximal passive interincisal opening increased to 45 mm. The persistence of restricted mouth opening despite neuromuscular relaxation, followed by an immediate increase after resection, supports a mechanical contribution of the ossified stylohyoid complex to the preoperative restriction, but does not establish the exact mechanism. Comparison with exceptionally long stylohyoid measurements reported in the literature requires careful interpretation because different authors have measured different anatomical structures. These have included the osseous styloid process alone, an ossified stylohyoid ligament, or the entire ossified stylohyoid chain [14,15,16,17]. Radiological measurements and measurements of surgically excised specimens should therefore be reported separately and interpreted according to the anatomical landmarks used. In the present case, the approximately 90-mm radiological measurement described the ossified stylohyoid chain extending from the skull base to the hyoid region; the residual ligamentous attachment to the lesser horn was identified at surgery. The combined length of the surgically removed fragments was 80 mm, and an approximately 10-mm proximal segment was intentionally retained. The imaging and specimen measurements describe different extents and measurement conditions and should not be treated as an exact arithmetic reconstruction of the original chain. Recent reports have described stylohyoid complexes exceeding 100 mm on imaging as well as an approximately 100-mm surgically excised ossified stylohyoid chain [18,19]. These observations further demonstrate that comparisons of extreme measurements are meaningful only when both the anatomical structure being measured and the measurement method are clearly specified (Table S1).

Table S1 provides selected illustrative reports and is not intended as a systematic or exhaustive review of the literature.

**Figure 4:**
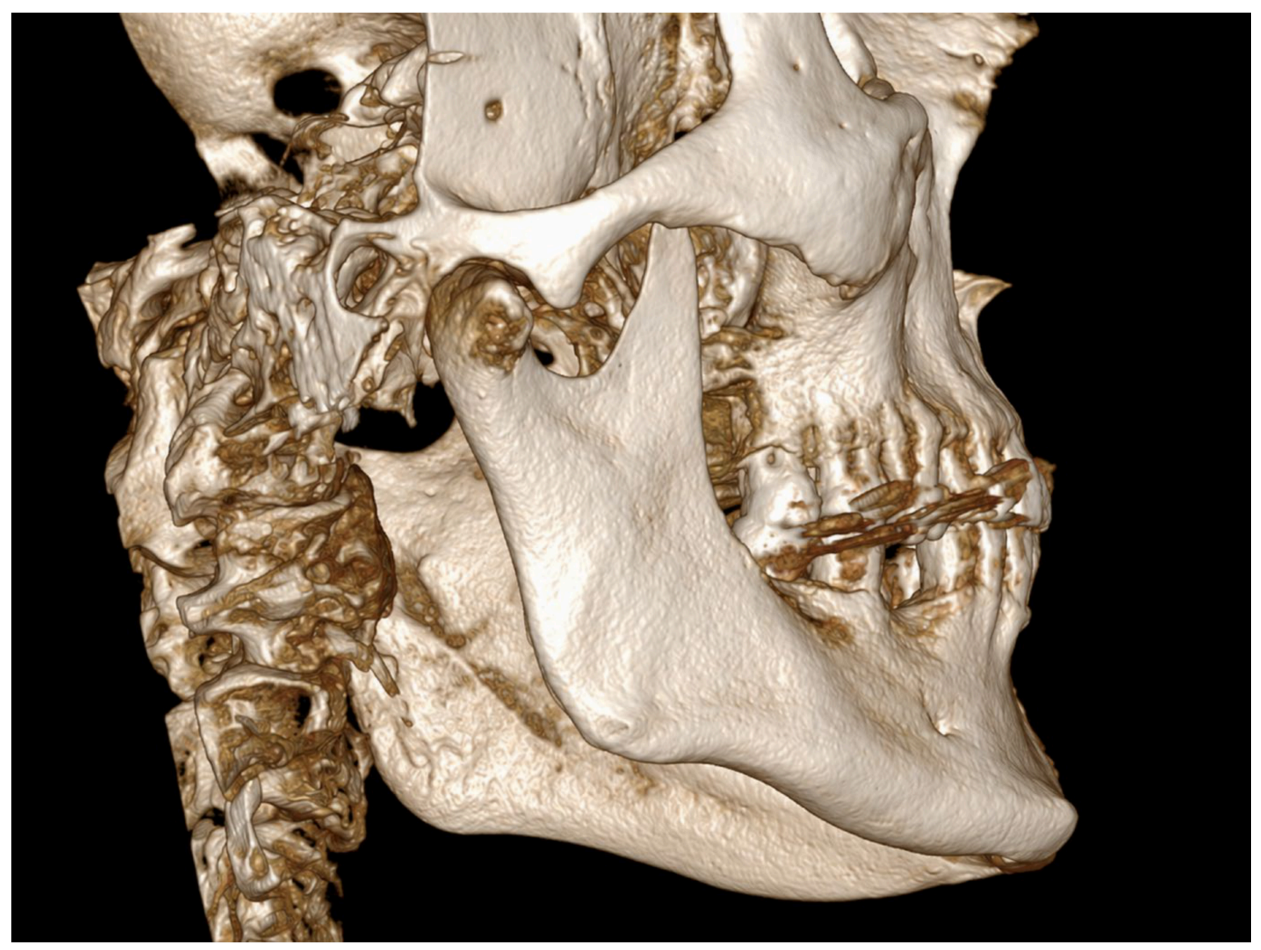
Postoperative cone-beam computed tomography obtained on the first postoperative day (Carestream CS 9600), with three-dimensional reconstruction of the right side, demonstrating postoperative appearance after resection of the ossified stylohyoid chain. Recovery was uneventful, with preserved facial nerve function and no sensory deficits. Pain decreased from 7/10 preoperatively to 3/10 on the first postoperative day and to 0/10 at six months. We initiated a structured 5-10-60 mouth-opening exercise protocol, as previously described by our group, on the first postoperative day [20]. Using a Heister mouth gag positioned between the upper and lower premolars or molars, the patient performed five exercise sessions per day, with ten mouth opening cycles during each session and a 60-s sustained stretch at each opening. The exercises were performed within a tolerable level of discomfort. This regimen was maintained for the first two weeks, followed by a gradual reduction in frequency to three sessions per day during the third and fourth weeks and subsequently to one session per day by the sixth to seventh postoperative week. Maximal interincisal opening was 25 mm preoperatively and 42 mm at six months. Restricted mouth opening is not a typical manifestation of Eagle syndrome, although it has been reported previously, including cases in which trismus or limited mouth opening improved following styloidectomy [21,22]. In the present case, maximal passive interincisal opening remained 25 mm despite neuromuscular blockade and increased immediately to 45 mm after resection, using the same operator and measurement method. At six months, maximal interincisal opening was 42 mm. These findings support a mechanical contribution of the ossified stylohyoid complex to the preoperative restriction. The six-month result reflects both surgical treatment and postoperative rehabilitation. Although a single case cannot establish a general causal relationship, restricted mouth opening may represent an uncommon manifestation of Eagle syndrome. In patients with otherwise unexplained limitation of mandibular movement, particularly when accompanied by cervicofacial pain or dysphagia, the stylohyoid complex should be considered as part of the differential diagnosis [6].

## Data Availability

The data presented in this study are available on request from the corresponding author. The data are not publicly available because they contain information that could compromise the privacy of the patient.

## Supplementary Materials

The following supporting information can be downloaded at https://www.mdpi.com/article/10.3390/diagnostics16193197/s1. Table S1. Selected reports of exceptionally long styloid processes and ossified stylohyoid complexes, with radiological and surgical specimen measurements reported separately.

## References

[1] Oztunc H., Evlice B., Tatli U., Evlice A. (2014). Cone-beam computed tomographic evaluation of styloid process: A retrospective study of 208 patients with orofacial pain. Head Face Med..

[2] Murtagh R.D., Caracciolo J.T., Fernandez G. (2001). CT findings associated with Eagle syndrome. AJNR Am. J. Neuroradiol..

[3] Langlais R.P., Miles D.A., Van Dis M.L. (1986). Elongated and mineralized stylohyoid ligament complex: A proposed classification and report of a case of Eagle’s syndrome. Oral Surg. Oral Med. Oral Pathol..

[4] Bargiel J., Gontarz M., Gasiorowski K., Marecik T., Wyszynska-Pawelec G. (2024). Outcomes of elongated styloid process syndrome treated with minimally invasive cervical styloidectomy (MICS): A single-center retrospective study. J. Clin. Med..

[5] Yavuz H., Caylakli F., Yildirim T., Ozluoglu L.N. (2008). Angulation of the styloid process in Eagle’s syndrome. Eur. Arch. Otorhinolaryngol..

[6] Badhey A., Jategaonkar A., Anglin Kovacs A.J., Kadakia S., De Deyn P.P., Ducic Y., Schantz S., Shin E. (2017). Eagle syndrome: A comprehensive review. Clin. Neurol. Neurosurg..

[7] More C.B., Asrani M.K. (2010). Evaluation of the styloid process on digital panoramic radiographs. Indian J. Radiol. Imaging.

[8] Bargiel J., Gontarz M., Marecik T., Szczurowski P., Gasiorowski K., Zapala J., Wyszynska-Pawelec G. (2023). Minimally invasive cervical styloidectomy in stylohyoid syndrome (Eagle syndrome). J. Clin. Med..

[9] Jayaraman M.V., Boxerman J.L., Davis L.M., Haas R.A., Rogg J.M. (2012). Incidence of Extrinsic Compression of the Internal Jugular Vein in Unselected Patients Undergoing CT Angiography. AJNR Am. J. Neuroradiol..

[10] Bargiel J., Gontarz M., Gasiorowski K., Marecik T., Wrona P., Zapala J., Wyszynska-Pawelec G. (2024). Stylohyoid chain syndrome (Eagle syndrome) in conjunction with carotid artery dissection: A case report of surgical treatment. Diseases.

[11] Li M., Sun Y., Chan C.C., Fan C., Ji X., Meng R. (2019). Internal jugular vein stenosis associated with elongated styloid process: Five case reports and literature review. BMC Neurol..

[12] Mejia-Vergara A.J., Sultan W., Kostas A., Mulholland C.B., Sadun A. (2022). Styloidogenic Jugular Venous Compression Syndrome with Papilloedema: Case Report and Review of the Literature. Neuroophthalmology.

[13] Fitzpatrick T.H., Lovin B.D., Magister M.J., Waltonen J.D., Browne J.D., Sullivan C.A. (2020). Surgical management of Eagle syndrome: A 17-year experience with open and transoral robotic styloidectomy. Am. J. Otolaryngol..

[14] Albayat A., Al Habeeb A., Jawad M. (2023). Dysphagia due to an extremely long styloid process: A case report of Eagle syndrome. Cureus.

[15] Kubikova E., Varga I. (2009). A case of extremely long styloid process without clinical symptoms and complications. Clin. Anat..

[16] Alaqeeli A.-A., Hamze A.K., Farag I. (2024). Eagles’ syndrome: Is it the longest styloid process length to date? A case report and an overview of the literature. Discov. Med..

[17] Panwar A., Keluskar V., Charantimath S., Lokesh Kumar S., Sridhar M., Jayapriya T. (2022). Bilateral elongated styloid process (Eagle’s syndrome): A case report and short review. Acta Otolaryngol. Case Rep..

[18] Li H., Zhang S.-Q., Zhang L., Liu H., Zheng H.-Y. (2025). Eagle’s syndrome with stylohyoid chain pseudoarthrosis and thyrohyoid ligament ossification: A case report and literature review. Medicine.

[19] Thapa G., Tao W.X., Jing L.Z., Jian Y. (2026). An uncommon extensive bilateral continuous ossification of the stylohyoid chain extending to the hyoid bone in Eagle’s syndrome: A case report. Cureus.

[20] Miaskiewicz J., Lato A., Gontarz M., Gasiorowski K., Marecik T., Wyszynska-Pawelec G., Bargiel J. (2025). Surgical treatment of mandibular coronoid process hypertrophy syndrome. J. Clin. Med..

[21] Aldelaimi T.N., Boskani S.W., Ali S.M., Mahmud P.K. (2017). Eagle syndrome: An unusual cause limited mouth opening and surgical management. J. Craniofac. Surg..

[22] Maki D., Okami K., Ebisumoto K., Sakai A. (2018). Chronological progression of an enlarged styloid process: A case report of Eagle syndrome. Case Rep. Otolaryngol..

