# Supplementary material for: Giant 90-mm Ossified Stylohyoid Chain Associated with Restricted Mouth Opening
Source: Diagnostics (Basel). 2026 Oct 1;16(19):3197. doi: 10.3390/diagnostics16193197 (PMC13664499; doi:10.3390/diagnostics16193197)
Supplement: Supplementary file 1 [file diagnostics-16-03197-s001.zip › diagnostics-4573571-supplementary.pdf]

Table S1. Selected reports of exceptionally long styloid processes and ossified stylohyoid complexes, with radiological and surgical specimen measurements reported separately.

Report	Structure reported	Radiological length	Specimen length	Surgery
Albayat et al., 2023 [14]	Reported as an elongated styloid process	149 mm	Not stated	Yes, extent not stated
Kubikova and Varga, 2009 [15]	Styloid process, bilateral	140 mm	Not applicable	No, asymptomatic
Thapa et al., 2026 [19]	Bilateral ossified stylohyoid complex; continuous left-sided chain extending to the hyoid bone	101.1 mm left; 80.4 mm right	Not reported	Yes; transcervical resection
Li et al., 2025 [18]	Completely ossified chain with pseudoarthrosis	Not stated separately	Approximately 100 mm, including ossified ligament, styloid process and part of the hyoid bone	Yes, excised en bloc
Present case	Unsegmented ossified chain extending to the hyoid region	90 mm; cross-section 17.5 × 10.5 mm	80 mm, in two fragments	Yes; approximately 10-mm proximal stump retained; distal ligament released
Alaqeeli et al., 2024 [16]	Styloid process, deformed, reaching greater horn of hyoid bone	85 mm	Not stated	Yes; postoperative imaging showed a 33 mm residual segment
Bargiel et al., 2024 [4]	Styloid process, longest of 84 operated cases	80 mm	Not stated	Yes
Panwar et al., 2022 [17]	Styloid process, bilateral	70.7 and 60.5 mm	Not applicable	No
